# Placental and fetal consequences of maternal selective serotonin reuptake inhibitor use: current evidence and unresolved questions

**DOI:** 10.3389/fphar.2026.1789010

**Published:** 2026-04-10

**Authors:** McKenna J. Crossen, Laura L. Hernandez

**Affiliations:** Department of Animal and Dairy Sciences, University of Wisconsin-Madison, Madison, WI, United States

**Keywords:** antidepressants, fetal health, placenta, pregnancy, selective serotonin reuptake inhibitor

## Abstract

Serotonin is a neurotransmitter critical for mood regulation, neurodevelopment, and diverse organ functions. It is synthesized from tryptophan through tightly regulated enzymatic pathways that maintain homeostasis, particularly during pregnancy. Selective serotonin reuptake inhibitors (SSRIs) are commonly prescribed across age groups to manage depression, anxiety, and other mental health conditions, with treatment tailored to individual needs. During pregnancy, SSRIs are widely prescribed or maintained to manage maternal health. Despite decades of research on SSRIs and pregnancy, there continues to be conflicting conclusions with some studies reporting beneficial effects on maternal and fetal health and outcomes, while others suggest potential risks. This review aimed to identify overall trends and evolution of our understanding of SSRI use and pregnancies from studies published from 1983–2025, including comparisons between placental roles of two major model organisms: mice and humans, serotonin biology, pharmacological properties, and prescription mechanisms of SSRIs, and the impact of SSRI use during pregnancy and the continued gaps in current research. Despite extensive research, there is a significant gap in understanding the safety and effects of SSRI use specifically in advanced maternal age pregnancies, as the age of first-time mothers continues to shift past 30 years old, highlighting a critical area for future research and clinical guidance.

## Introduction

Depression and Anxiety are prevalent among women of all ages, including reproductive age, and can have significant consequences for both maternal and fetal health (Upadhyaya et al., 2023; Lebin and Novick, 2022). Selective Serotonin Reuptake Inhibitors (SSRIs) are widely prescribed to manage a variety of different conditions during pregnancy due to their continued efficacy and importance for maternal health (Amit et al., 2024). Serotonin, a neurotransmitter critical for mood regulation, neurodevelopment, and multiple organ systems (Bamalan et al., 2021), is tightly regulated during pregnancy to support maternal and fetal serotonin exposure, thereby influencing fetal development and maternal health (Rosenfeld, 2020).

Despite extensive research, the effects of SSRI use during pregnancy remain conflicting. Some studies suggest benefits for maternal mental health and favorable outcomes, while others suggest potential risks for both mother and offspring (Levy et al., 2020; Hutchison et al., 2018; Domar et al., 2013).

Most research to date has focused on general pregnancy populations, leaving a critical gap regarding advanced maternal age (AMA) pregnancies (pregnancies in mothers 35≤). Further, there is a growing trend of older nulliparous mothers (≥30 years) delaying childbearing, which has become increasingly common (Brown et al., 2025), and the general increase in SSRI use throughout all age groups (Frangou et al., 2023). Therefore, continued research on how SSRIs and AMA affect maternal, fetal, and placental outcomes is critical for safe clinical management, especially in this age range.

This is a narrative, hypothesis-generating review paper based on research spanning from critical foundational work dating back to 1983 to studies from 2000–2025, examining serotonin biology, SSRI pharmacology, placental roles in humans and mice, and maternal and fetal outcomes. Human and mouse data are presented to emphasize convergent patterns and outstanding mechanistic questions, while also acknowledging species-specific differences that constrain direct translational inference. Special attention is given to the gaps in knowledge surrounding SSRI use in AMA pregnancies, pregnancies in mothers ≥35 years old, highlighting key areas for future research and evidence-based guidance in clinical practice.

## Serotonin biology

Serotonin, or 5-hydroxytryptamine (5-HT), is a neurotransmitter that plays an important role throughout the entirety of the human body-regulating a variety of homeostatic activities, including behavior (Siegel and Crockett, 2013), mood (Kanova and Kohout, 2021), memory (Coray and Quednow, 2022), immune reactions (Nakamura et al., 1999; Duerschmied et al., 2013; Wu et al., 2019), and GI activity (Bamalan et al., 2021; Baković et al., 2021; Mohammad-Zadeh et al., 2008; Moncrieff et al., 2023). Serotonin’s role can be divided into two major categories: roles within the central nervous system and roles within the peripheral nervous system, based on the location of 5-HT synthesis (Kanova and Kohout, 2021). Within the CNS, the serotonin neurons in the brainstem project upward toward the brain in an organized system that terminates in the cortical, limbic, midbrain, and hindbrain regions (Berger et al., 2009). This is what allows serotonin to regulate the neural circuitry responsible for behavior and emotion (Roth et al., 2004; Canli and Lesch, 2007; Airan et al., 2007). Within the peripheral system, serotonin is critical for GI motility, triggering peristalsis and secretions (Gershon, 2012), vasoconstriction and vasodilation of the cardiovascular system (Watts et al., 2012), pain signaling, and sensory function (Sommer, 2004), immune cell reactions (Duerschmied et al., 2013), and functioning as both an autocrine and paracrine signaling molecule alongside its role as a neurotransmitter.

### Serotonin synthesis and storage

**FIGURE 1:**
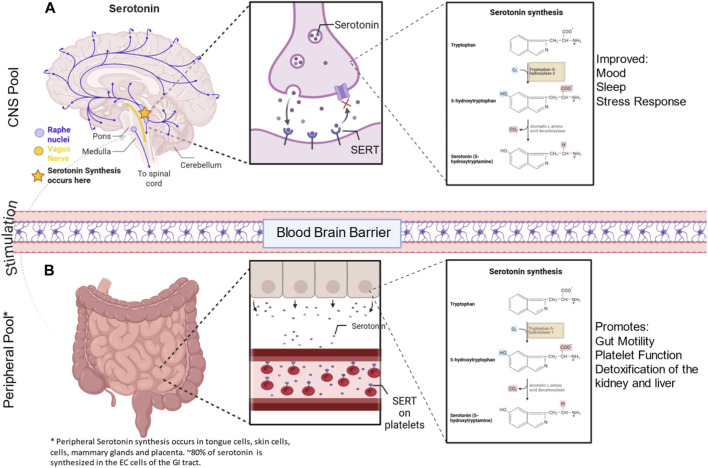
Serotonin Pools and SERT action throughout the body. Serotonin is synthesized in two major pools: The central nervous system (CNS) pool and the Peripheral Pool. **(A)** In the CNS pool, the raphe nuclei, specifically the rostral raphe nuclei, which project to the forebrain and the dorsal raphe nucleus, synthesize serotonin. Serotonin is synthesized from tryptophan to 5-hydroxytryptophan (5-HT) *via* tryptophan-5-hydroxylase-2 (TPH2), which is only located in the brain. When SSRIs are used, they block the reuptake of serotonin in the synaptic cleft, allowing it to remain available, resulting in improved mood, sleep, and stress responses. **(B)** In the peripheral pool, 80% of the entire body’s serotonin is synthesized in the enterochromaffin cells of the GI tract, it also occurs in the tongue cells, skin cells, mammary glands, and placenta. Serotonin synthesis occurs similarly to that in the CNS pool; however, tryptophan is converted to 5-HT by tryptophan hydroxylase-1 (TPH1). When SSRIs are used, they will promote gut motility, platelet function, detoxification of the kidney and liver, regulate milk production, and provide a source of serotonin to the developing fetus. Stimulation from the EC cells of the peripheral pool results in the vagus nerve being stimulated, resulting in gut-brain communication. Figure was created using BioRender at biorender.com.

Serotonin is predominantly synthesized in the raphe nuclei of the brain (Figure 1A) and the enterochromaffin cells (EC) of the intestinal mucosa (Figure 1B) (Owens and Nemeroff, 1994; Masand and Gupta, 1999); however, it can also be synthesized in other tissues, including pulmonary neuroendocrine cells (Archambault and Delaney, 2023), tongue cells (Pan et al., 2018), and Merkel cells of the skin (Hoffman et al., 2018), in the mammary gland to regulate milk secretion and epithelial lactogenic conditions (Sheftel and Hernandez, 2020), and in the placenta to provide an early supply of serotonin to the developing fetus (Perić et al., 2022). Serotonin utilizes a two-step enzymatic pathway to convert tryptophan to 5-HT. The first step is the hydroxylation of tryptophan, in which tryptophan hydroxylase (TPH), the rate-limiting step, converts tryptophan to 5-Hydroxytryptophan (5-HTP) (Fernstrom, 2016) (Figure 1). Following this step, 5-HTP is decarboxylated via aromatic L-amino acid decarboxylase (AAAD) to produce serotonin (5-HT) (Hasegawa et al., 2010). Approximately 80%–90% of the body’s serotonin is synthesized by EC cells, stored in platelets, and transported through the bloodstream to act on various tissues outside the CNS. This phenomenon occurs in part because 5-HT cannot cross the blood-brain barrier. Serotonin is hydrophilic and polar (Frazer and Hensler, 2021), and at the physiological pH of ∼7.4 at the blood-brain barrier, it is fully protonated at the amine, resulting in a +1 charge (Omidyan et al., 2017; Kanova and Kohout, 2021). Serotonin reuptake transporters (SERT) are located on numerous tissues throughout the body (Gill et al., 2008), including on the membrane of the platelets (Kanova and Kohout, 2021), in addition to neurons in the CNS. This allows serotonin to act on various surrounding tissues and organ systems by interacting with 5-HT receptors (Berger et al., 2009). Previous research has shown that all 14 serotonin receptor subtypes found in the brain are also present throughout the body and promote different metabolic functions depending on the site of the receptor (Kanova and Kohout, 2021). Serotonin signaling is critical for functions outside the central nervous system, including modulation of gut motility and secretion in the GI tract (Zhou and Verne, 2022). Further, 5-HT promotes vasoconstriction and platelet aggregation (Mercado and Kilic, 2010), regulates pulmonary vascular tone and smooth muscle proliferation (Haub et al., 2009), modulates development (Haub et al., 2009), and metabolism (Chen et al., 2012). Finally, a major role of serotonin signaling is detoxification in organs such as the kidneys (Kaur and Krishan, 2020) and liver (Ruddell et al., 2008), and the clearance of serotonin to prevent vasoconstriction of the placental vasculature (Hadden et al., 2017; Ellenberger et al., 2025; Karahoda et al., 2020; Staud et al., 2023). Because serotonin functions throughout the body, SERT is critical for the reuptake and degradation of serotonin in multiple cell types, thereby maintaining balanced control of serotonin’s diverse effects.

**FIGURE 2:**
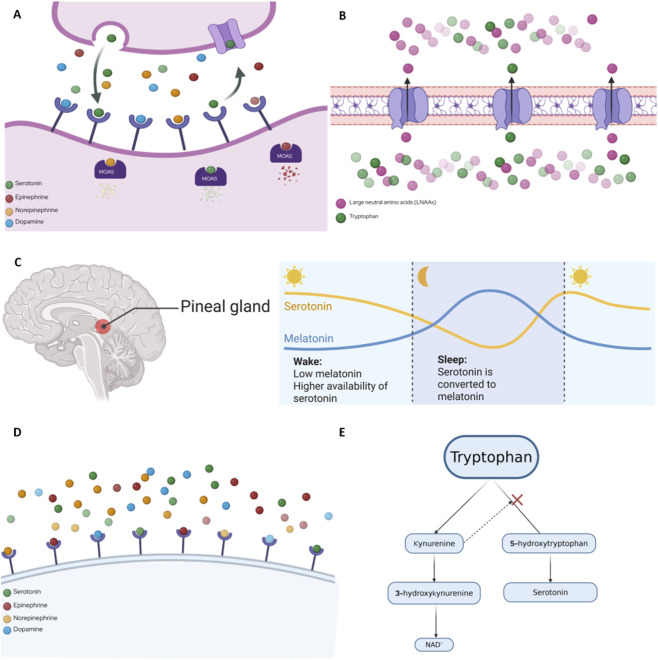
Summary of Serotonin (5-HT) Competition. **(A)** Serotonin Reuptake is naturally inhibited based on vesicular monoamine transporter (VMAT) availability. Other monoamines, such as dopamine, norepinephrine, and epinephrine, can restrict this reuptake by attaching to the VMAT before 5-HT does. **(B)** Other natural limitations are the decrease in 5-HT synthesis based on tryptophan availability. This restriction occurs at the blood-brain barrier, where other large neutral amino acids (leucine, isoleucine, valine, tyrosine, and phenylalanine) use the LAT1 transporter, reducing tryptophan entry into the brain. **(C)** Based on the circadian rhythm and wake vs. sleep cycles in which during sleep 5-HT is converted to melatonin, decreasing 5-HT availability at night. **(D)** Other monoamines such as dopamine, norepinephrine, and epinephrine can restrict 5-HT metabolism by monoamine oxidase (MAO) *via* attaching to VMAT and reducing 5-HT storage and release. **(E)** 5-HT synthesis can be reduced as metabolites from the kynurenine pathway can divert tryptophan away from 5-HT synthesis, especially during systemic inflammation. Figure was created using BioRender at biorender.com.

Serotonin has a relatively short biological half-life, typically 1–2 min in circulation, due to rapid uptake by SERT and enzymatic degradation by monoamine oxidase (MAO) (Fraer and Kilic, 2015; Daws, 2009). This short half-life reflects the tight regulation of serotonin in both the central and peripheral systems (Berger et al., 2009). Once released, serotonin is reabsorbed by SERT within ∼1–3 s (Dankoski and Wightman, 2013) and stored in platelets or degraded by MAO-A (Prah et al., 2020). Serotonin is then degraded into 5-hydroxyindoleacetic acid (5-HIAA), which is then excreted from the body, preventing prolonged receptor activation (Bamalan et al., 2021; Bortolato et al., 2010). In contrast, pharmacological agents such as selective serotonin reuptake inhibitors (SSRIs) extend serotonin’s functional half-life by blocking its reuptake, leading to increased synaptic concentrations and sustained signaling (Owens and Nemeroff, 1994; Blier and de Montigny, 1999). Understanding this kinetic profile is essential for interpreting both physiological serotonin activity and the pharmacodynamic effects of serotonergic drugs (Stahl, 2021). Together, reuptake and breakdown help maintain balanced serotonin levels in the brain and body (Figure 2).

### Serotonin in blood

Platelets serve as the primary peripheral reservoir of 5-HT in the blood, although they do not synthesize it themselves. They acquire 5-HT from plasma *via* SERT and store it in dense granules, releasing it upon activation (Duerschmied et al., 2013). Released platelet 5-HT contributes to hemostasis (Fraer and Kilic, 2015), vasoconstriction (Brenner et al., 2007), inflammatory responses (Mauler et al., 2019), regulation of clot formation, and vascular tone (Maclean et al., 2019). Because platelets mirror systemic serotonergic activity, platelet serotonin levels are often used as a peripheral biomarker of serotonergic function, and alterations have been linked to psychiatric disorders (Owens and Nemeroff, 1994; Giurgiuca et al., 2016; Koudouovoh-Tripp and Sperner-Unterweger, 2012; Gunay et al., 2025; Zhuang et al., 2018), cardiovascular disease (Williams et al., 2019), and platelet dysfunction (Yun et al., 2016; Gianazza et al., 2020). Importantly, it should be noted that 5-HT does exist in platelets, serum, and plasma. In plasma, the average 5-HT concentrations are 1–5 ng/mL, with a circulating plasma half-life of 1–2 min due to 5-HT degradation by MAO-A in the endothelial cells and liver (Brenner et al., 2007). Average 5-HT concentrations in the platelets is 0.2-2ug per 10^9^ platelets, with a half-life of 4–5 days (Li et al., 1997), due to the lifespan of platelets (Bismuth-Evenzal et al., 2012). Previous research found that platelet 5-HT content was approximately 66% lower in patients who were on extended exposure to SSRIs (6–108 months) (Bismuth-Evenzal et al., 2012). Finally, in serum, the average 5-HT concentration is 70–270 ng/mL, with a half-life of approximately 1–2 min (Moroianu et al., 2022). Serum 5-HT concentrations are measured from clotted blood because platelets release 5-HT during clotting. Unfortunately, research on the impact of SSRIs on these serotonin reservoirs is not well known due to difficulties in collecting data or inconsistencies in study results. These differing concentrations are important, especially when interpreting 5-HT levels in patients on SSRIs, as platelet levels will be much lower due to reduced systemic serotonin reuptake (Bismuth-Evenzal et al., 2012).

### Pools of serotonin storage

Serotonin exists in two separate pools. There is a central serotonin pool in the CNS and a peripheral 5-HT pool throughout the rest of the body (Kanova and Kohout, 2021). In the CNS, 5-HT is synthesized by TPH2, which is exclusively expressed in neuronal cells, specifically serotonergic neurons, in the raphe nucleus of the midbrain (Berger et al., 2009). In the CNS, 5-HT acts as a neurotransmitter affiliated with the stress response, sleep and mood (Kanova and Kohout, 2021) (Figure 1A). In the peripheral 5-HT pool, TPH1 synthesizes serotonin, which acts as a hormone that affects gut motility and platelet function (McKinney et al., 2005). Interestingly, both pools communicate with the vagus nerve to form the gut-brain axis, which serves as a communication network between the CNS and the peripheral system (Hwang and Oh, 2025) (Figure 1B). This is critical because the two pools cannot cross the blood-brain barrier due to the polarity of 5-HT, which prevents it from passing through the lipid-based blood-brain barrier (Kanova and Kohout, 2021).

### Serotonin receptor competition

Serotonin has no direct natural competitive inhibitors at its receptors, but several naturally occurring competitors regulate its activity. Other monoamines, such as dopamine, norepinephrine, and epinephrine, can compete with 5-HT for uptake by the vesicular monoamine transporter (VMAT) (Figure 2A) and for metabolism by monoamine oxidase (MAO), thereby reducing 5-HT storage and release (Amara and Arriza, 1993; Uhl and Johnson, 1994) (Figure 2D). Serotonin synthesis also depends on tryptophan availability, and tryptophan must compete with other large neutral amino acids (LNAAs) such as leucine, isoleucine, valine, tyrosine, and phenylalanine for transport across the blood–brain barrier *via* the LAT1 transporter (Kuhn et al., 2020); when more LNAAs are present, less tryptophan enters the brain, and 5-HT production falls (Fernstrom, 1994) (Figure 2B). Additionally, endogenous metabolites from the kynurenine pathway can reduce 5-HT synthesis by diverting tryptophan away from the 5-HT pathway, an effect that is especially pronounced during inflammation (Schwarcz and Stone, 2017) (Figure 2E). Finally, 5-HT is also converted into melatonin in the pineal gland, thereby decreasing 5-HT availability at night (Hardeland and Poeggeler, 2012) (Figure 2C). In summary, while the body does not produce true natural competitive inhibitors that directly block 5-HT receptors, 5-HT’s action is modulated through competition at the levels of transport, metabolism, and precursor availability.

## Selective serotonin reuptake inhibitors (SSRIs)

### How do SSRIs work?

Selective serotonin reuptake inhibitors are a class of antidepressants that increase 5-HT levels in the synaptic cleft by blocking the SERT, which normally reabsorbs 5-HT into the presynaptic neuron (Edinoff et al., 2021). By preventing reuptake, SSRIs enhance serotonergic neurotransmission (Stahl, 1998), leading to improved mood (Michely et al., 2020), reduced anxiety (Garakani et al., 2020), and the regulation of other functions controlled by 5-HT, such as sleep, appetite, and pain perception (Boschloo et al., 2023). Over time, chronic SSRI use also induces downregulation of presynaptic autoreceptors (5-HT1A) and adaptive changes in postsynaptic receptor sensitivity, which contribute to their therapeutic effects (Turcotte-Cardin et al., 2019). The goal is to maximize antidepressant efficacy while minimizing side effects, such as sedation, anticholinergic effects, and cardiotoxicity, commonly observed in patients who utilize tricyclic antidepressants (TCA).

### Types of SSRI’s and how they differ

Selective serotonin reuptake inhibitors primarily differ in half-life, metabolism, receptor selectivity, and side-effect profile. Commonly prescribed SSRIs share a mechanism of action by blocking SERT but differ in pharmacokinetics, side effects, and interaction profiles (Table 1).

**TABLE 1:** Summary of selective serotonin reuptake inhibitors (SSRIs): approved and off-label indications, pharmacokinetic parameters, common side effects, and clinical implications.

Name	On label prescription	Off- label prescription	Peak concentration	Half-life	Side effects	Implications	Citations
Sertraline	MDD, OCD, PD, PMDD, social anxiety disorder, PTSD	PTSD, GAD, *Postartum* depression, premature ejaculation, ED	4–10 h	24–32 h	GI upset, Dopamine Effect	Easier to taper and less rigid scheduling	(Huddart et al., 2020; Sanchez et al., 2014; ZOLOFT, 2020)
Citalopram	MDD	Anxiety, Agitation (dementia)	1–4 h	35 h	QT prolongation	Most Selective SSRI	(Sangkuhl et al., 2011; Sanchez et al., 2014; Landy et al., 2023; Bousman et al., 2023; LLC AP, 2023)
Paroxetine	MDD, OCD, PD, GAD, social anxiety disorder, PTSD, PMDD	Premature Ejaculation, Menopause symptoms	4 h	24 h*	Neurological, GI side effects	Higher Withdrawal	(Tang and Helmeste, 2008; Corp, , 2021)
Fluvoxamine	OCD	social anxiety disorder, PTSD, depression, excoriation disorder	capsules: 2–8 h; tablets 4–12 h	12–15 h	Increased drug interactions	Reduced Clearance due to Interactions	(Goodman et al., 1997; van Harten, 1995; Par Pharmaceutical, 2017)
Fluoxetine	MDD, OCD, Bulimia Nervosa, PD, PMDD	GAD, social anxiety disorder, PTSD, Augmentation, Pediatric Depression	6–8 h	2–4 days	Nausea, diarrhea, headache, insomnia, SD	Long half-life → lower withdrawal risk, slower steady state; strong CYP2D6 inhibitor	(Perez-Caballero et al., 2014; Hiemke and Härtter, 2000; Edgomont Pharmaceuticals, 2017)
Escitalopram	MDD, GAD	social anxiety disorder, PD, OCD, PTSD	4–6 h	27–32 h	GI upset, headache, decreased libido	Lower withdrawal risk, similar to citalopram	(Sanchez et al., 2014; Landy et al., 2023; Allergen, 2017)

Abbreviations: ED, eating disorder; GAD, generalized anxiety disorder; OCD, obsessive-compulsive disorder; PD, panic disorder; PTSD, post-traumatic stress disorder; social anxiety disorder, SD, sexual dysfunction.

*Fluoxetine* (Prozac ®), the first SSRI approved in 1987, was discovered by Eli Lilly scientists who were searching for compounds that selectively inhibited 5-HT reuptake without affecting norepinephrine or dopamine (Perez-Caballero et al., 2014). Fluoxetine, which is primarily prescribed for major depressive disorder (MDD), obsessive compulsive disorder (OCD) and Bulimia Nervosa while it is also prescribed for a variety of other mental health disorders, including social anxiety disorder, generalized anxiety disorder, premenstrual dysphoric disorder (PMDD), post-traumatic stress disorder (PTSD), panic disorder, and augmentations. It has the longest half-life (2–4 days; the active metabolite, norfluoxetine, up to 2 weeks), which minimizes withdrawal risk but strongly inhibits cytochrome P450 2D6 (CYP2D6). Cytochrome P450 2D6 has been shown to be critical in the metabolism of approximately 20% of commonly used pharmaceuticals (Tayl et al., 2020; Stahl, 2021; Hiemke and Härtter, 2000). Previous research determined CYP2D6 inhibition results in elevated levels of ingested pharmaceutical drugs circulating throughout the body (Tayl et al., 2020). After the development of fluoxetine, researchers used observations and data from its use to optimize selectivity, half-life, and tolerability, leading to the development of sertraline, citalopram, escitalopram, and fluvoxamine.

*Sertraline* (Zoloft®) is commonly prescribed to treat a myriad of psychiatric disorders, including depression, panic disorder, seasonal affective disorder (SAD), PTSD, PMDD, postpartum depression, and generalized anxiety (Huddart et al., 2020), and often produces gastrointestinal upset, but is relatively safe in cardiac patients and has mild dopamine transporter effects that may be energizing (Gorman et al., 2000; Kent et al., 1998; Preskorn, 1997). Sertraline reaches peak concentrations approximately 4–10 h after ingestion, with a half-life of 24–32 h (Huddart et al., 2020).

*Citalopram* (CeleXA®) is commonly prescribed for MDD, anxiety and agitation in dementia patients and has few drug interactions but carries a dose-dependent risk of QT prolongation (McClelland and Mathys, 2016). Citalopram has a shorter time to peak concentration, approximately 1–4 h, but a longer half-life than sertraline, approximately 35 h after administration (Sangkuhl et al., 2011).

Escitalopram (Lexapro®), is the S-enantiomer of citalopram, is better tolerated and among the most selective SSRIs (Sanchez et al., 2014). Being used for similar mental health disorders as citalopram, escitalopram is used for generalized anxiety disorder (GAD) and MDD. Escitalopram has a shorter time to peak concentration, approximately 4–6 h, with a half-life of 32 h, which is shorter than citalopram; however, escitalopram is a purer form of citalopram, therefore the half-life is shorter (Sanchez et al., 2014; Landy et al., 2023). Importantly, there is a lower risk of prolonged QT compared with citalopram (Hasnain et al., 2013).

*Paroxetine* (Paxil®) is used to treat MDD, OCD, panic disorder (PD), GAD, SAD, and PTSD. Paroxetine has the shortest half-life, resulting in significant withdrawal symptoms, and is linked to weight gain, sedation, sexual dysfunction, and anticholinergic effects, making it less ideal in elderly patients (Stahl, 2021; Hiemke and Härtter, 2000). Paroxetine will reach peak concentrations approximately 4 h after oral administration (Sangkuhl et al., 2011). Paroxetine has a terminal elimination half-life of about 24 h, but there is variation due to enzymatic activity, which is attributed to different genetic polymorphisms of the SERT receptors (Foster and Goa, 1997). Variations include CYP2D6, which reduces drug metabolism (Hole et al., 2023); SLC6A4, which decreases receptor expression (Bousman et al., 2023); and increased HTR2A sensitivity, leading to increased signaling by paroxetine (Bousman et al., 2023). Finally, impairment of ABCB1, a gene associated with predicting depression remission following treatment (Simoons et al., 2020), may restrict paroxetine transport and concentration across the blood-brain barrier, thereby limiting central nervous system exposure (Kato et al., 2008).

*Fluvoxamine* (Luvox®) is less commonly prescribed for depression and more commonly prescribed for PD, SAD, and PTSD, but is particularly effective in obsessive–compulsive disorder; however, it is a strong CYP1A2 and CYP2C19 inhibitor, increasing drug interaction risk (Hiemke and Härtter, 2000; Goodman et al., 1997). Fluvoxamine also acts as a sigma-1 agonist, which allows it to modulate stress responses in cells and contributes to its therapeutic effects (Ahmed et al., 2025). The inhibition of these receptors will result in a reduction in fluvoxamine clearance and also prevent the metabolism of other drugs, including diazepam (Perucca et al., 1994), clopidogrel (Hirsh-Rokach et al., 2015), and theophylline (Hirsh-Rokach et al., 2015; Hemeryck and Belpaire, 2002). Interestingly, Fluvoxamine peak concentrations depend on the type of tablet or capsule, with peak concentrations achieved approximately 2 or 8 h after administration, while enteric-coated tablets reach peak within 4–12 h after administration (van Harten, 1995). Fluvoxamine has a terminal elimination half-life of 12–15 h after initial administration (van Harten, 1995).

Sertraline and fluoxetine are the most prescribed during pregnancy (Lebin and Novick, 2022). Overall, SSRIs represent a major advancement in antidepressant therapy, offering selective 5-HT modulation with improved tolerability; however, individual agents differ in pharmacokinetics, side-effect profiles, and pregnancy safety, which should guide clinical selection.

## Pharmacokinetic changes during pregnancy

During pregnancy, the body will go through a variety of changes to ensure it can support the developing fetus while also remaining in a homeostatic balance, including pharmacokinetic changes that impact how different drugs are absorbed. Gastric emptying and intestinal motility will slow, resulting in an increase in gastric pH, which alters drug absorption and delays peak concentration (Pariente et al., 2016). Alternatively, plasma volume increases, while plasma albumin and other binding proteins decrease, leading to an increase in the volume of distribution of many drugs (Costantine, 2014). Hepatic metabolism changes in an enzymatic manner with several enzymes (CYP3A4, CYP2D6, CYP2C9) increasing, resulting in an increase in clearance of their substrates, while others, like CYP12A2 AND CYP2C19, decrease, resulting in an increased exposure to their substrates (Pariente et al., 2016). Renal blood flow and glomerular filtration rate rise, causing an increased clearance and possibly lower concentration of renally eliminated drugs (Anderson, 2005). While these adaptations are helpful for pregnancy, they also make oversight and adjustments necessary for therapeutic monitoring.

## The placenta: roles and differences in mice and humans

### Major roles of the placenta

The placenta is a dynamic organ that forms from both maternal and fetal tissues, establishing the interface for nutrient, gas, and waste exchange while producing hormones critical for pregnancy (Cross, 2006). The placenta will transfer oxygen and essential nutrients, including glucose, amino acids, fatty acids, and vitamins, from the maternal blood while removing carbon dioxide and metabolic waste from fetal blood (Bloom et al., 2022). The placenta also acts as a selective barrier, preventing many pathogens from reaching the fetus while allowing the transfer of maternal antibodies (IgG) for passive immunity (Tizard, 1982; Palmeira et al., 2012). Importantly, the placenta will produce hormones including human chorionic gonadotropin (hCG) (Orendi et al., 2011), progesterone (Orendi et al., 2011), estrogen (Orendi et al., 2011), and human placental lactogen (hPL) (Rassie et al., 2022), which are critical in the maintenance of the uterine lining and maternal metabolism, ultimately impacting the fetal growth. It is important to note that the mother and fetus have two separate circulatory systems that exchange with one another (Burton and Jauniaux, 2023).

### Development

The human placenta develops shortly after fertilization, beginning when the blastocyst attaches to the uterine lining around days 6–7 (Salkeld et al., 2008). The outer trophoblast differentiates into the cytotrophoblast and the invasive syncytiotrophoblast (James et al., 2012), which embeds the embryo and initiates maternal blood flow (Hanna, 2020). Around days 12–16, cytotrophoblast cells form chorionic villi, which develop fetal blood vessels, establishing the fetal-maternal interface (Huang et al., 2023). By weeks 3–4, fetal circulation connects to the umbilical cord, allowing nutrient and gas exchange. By the end of the first trimester, the placenta is fully functional, producing hormones that support pregnancy (Burton and Jauniaux, 2023). Throughout gestation, the villi remodel to optimize exchange, while the placenta continues to grow and adapt to fetal needs (Kingdom et al., 2000).

### Mouse placenta

Although the primary focus of this review is the impact of SSRIs on human placental and offspring health, much of the essential research is initially conducted in mouse models before clinical translation. Therefore, a comprehensive understanding of mouse placental development is essential for interpreting these studies and their relevance to human health. Mouse placental development begins around embryonic day (E) 3.5, when the trophectoderm (TE) of the blastocyst gives rise to all trophoblast lineages (Simmons et al., 2007). By E4.5, the blastocyst attaches to the uterine epithelium, and TE cells differentiate into invasive trophoblast giant cells that remodel maternal capillaries, while non-invasive TE cells form the ectoplacental cone (EPC) (Simmons et al., 2007). Between E7.5 and E10.5, the chorion fuses with the embryonic allantois to form the chorioallantois placenta and establish fetal circulation. The placenta then differentiates into three main zones: the labyrinth (maternal–fetal exchange), the spongiotrophoblast layer (endocrine and structural support), and the trophoblast giant cell layer (invasive and hormone-secreting) (Watson and Cross, 2005). By E16.5, the placenta reaches full maturation and maintains fetal growth until parturition at E19–E21 (Rossant and Cross, 2001).

### Layers of the placenta

In humans, on the maternal side, the placenta attaches to the decidua basalis (Guttmacher et al., 2014), which is derived from the endometrium (James et al., 2012; Hanna, 2020). The decidua basalis not only anchors the placenta but also contributes to the formation of maternal blood sinuses, which supply oxygen and nutrients to the developing fetus (Macnab, 2025; Mori et al., 2016).

From the fetal side, the placenta is primarily composed of chorionic villi, which are branching structures that invade the maternal tissue. Each villus contains fetal blood vessels and a connective tissue core, serving as the main site of maternal-fetal exchange (Huang et al., 2023). Surrounding these villi is the trophoblast, which initially forms as a multilayered structure. The cytotrophoblast, an inner layer of mononuclear cells, provides structural support and proliferative capacity, generating new trophoblast cells throughout gestation (Lawless et al., 2023). The syncytiotrophoblast, a multinucleated outer layer, is in direct contact with maternal blood and facilitates nutrient and gas exchange while producing critical hormones such as hCG and PL (Kolahi et al., 2017). Between the trophoblast and fetal capillaries lies the basement membrane and embryonic connective tissue, which provide structural support and mediate selective permeability (Burton and Jauniaux, 2023). The innermost layer, the fetal capillary endothelium, forms the walls of fetal blood vessels that transport oxygenated blood and nutrients to the fetus and carry waste products back toward the placenta (Guttmacher et al., 2014).

Throughout development, the placental barrier—composed of these layers—undergoes significant changes. Early in gestation, the barrier is thick, with both cytotrophoblast and syncytiotrophoblast layers that help protect the fetus from pathogens (Ding et al., 2022). As pregnancy progresses, the cytotrophoblast layer thins or becomes discontinuous in many areas, reducing the diffusion distance and increasing the efficiency of nutrient and gas exchange to meet the growing metabolic demands of the fetus (Sapehia et al., 2021). Simultaneously, the syncytiotrophoblast continues hormone production and maintains the interface with maternal blood (Sapehia et al., 2021). This coordinated development ensures both protection and efficient support for fetal growth from early embryogenesis through term.

In the mouse placenta, the barrier that separates maternal blood from fetal blood within the functional exchange region known as the labyrinth is composed of a characteristic trichorial arrangement of trophoblast-derived cells and fetal endothelium (Simmons et al., 2008). As maternal blood flows through the labyrinthine maternal sinusoids, it is first juxtaposed with a discontinuous layer of sinusoidal trophoblast giant cells (S-TGCs), which line the maternal blood spaces but do not form a complete continuous barrier (Panja and Paria, 2021). Immediately beneath this, two continuous layers of multinucleated syncytiotrophoblast cells—designated syncytiotrophoblast layer I (SynT-I) and syncytiotrophoblast layer II (SynT-II)—extend around the fetal capillaries and form the major trophoblast component of the interhaemal membrane (Simmons et al., 2008). Finally, on the fetal side of the barrier, fetal endothelial cells line the capillaries through which fetal blood circulates, completing the separation between the two circulations (Simmons et al., 2007). In summary, the primary exchange interface in the mature mouse placenta consists of three continuous cellular layers (the two syncytiotrophoblast layers and fetal endothelium), with the S-TGCs contributing to the maternal-facing side but not creating a continuous layer themselves; this arrangement maximizes surface area for efficient nutrient, gas, and waste exchange while maintaining separation of the maternal and fetal blood supplies (Simmons et al., 2008).

### Serotonin supply during pregnancy

**FIGURE 3:**
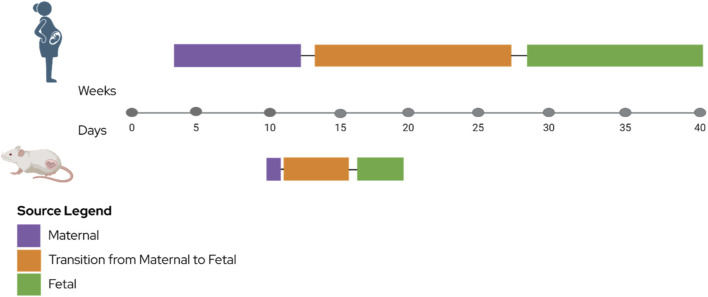
Summary of Serotonin Source during Gestation. Schematic timeline depicting the principal sources of serotonin (5-HT) throughout human and mouse development. During early gestation, maternal supply constitutes the primary source of 5-HT (purple). In mid-gestation, 5-HT production shifts from maternal to fetal sources as the brainstem develops, while placental contribution remains substantial (orange). In late gestation, the fetal brain serves as the predominant source of 5-HT (green). Human gestational weeks (top) and corresponding mouse embryonic days (bottom) are indicated. Figure created in BioRender, biorender.com.

During mammalian development, 5-HT regulates cell survival in early stages, with Tph2 and SERT expression detected as early as day 4 in humans (Basu et al., 2008; Bonnin and Levitt, 2011; Romero-Reyes et al., 2021). Placenta 5-HT is the main source of 5-HT for fetal brain development (Bonnin and Levitt, 2011; Côté et al., 2007), heart development (Romero-Reyes et al., 2021), and disruption to the cycle has been shown to contribute to future mental health disorders (Suri et al., 2015) (Figure 3). During the developmental timeline, gestational weeks 4–12 in humans and E10 in mice, the fetus relies on the mother to produce 5-HT (Bonnin and Levitt, 2011; St-Pierre et al., 2016). In the 2^nd^ trimester in humans, and E10.5-E15.5 in mice, the 5-HT supply transitions from maternal to fetal source as the brain stem is formed, relying on the placental supply to continue for proper embryonic development (Rosenfeld, 2020). Finally, in the 3^rd^ trimester in humans and E16.5 in mice, 5-HT will be used from the fetal brain (Bonnin and Levitt, 2011).

### Impact of placental development issues

If the placenta develops incorrectly, there are severe implications for both mother and fetus. If shallow trophoblast invasion occurs, spiral-artery remodeling will be altered, resulting in uteroplacental circulation in a high-resistance state. This ultimately can result in maternal vascular malperfusion and hypoxia (Lechner et al., 2023; Frederiksen et al., 2018; Stanek, 2023; Parks, 2015). The presence of hypoxia and malperfusion has strong associations with fetal growth restriction (FGR), preeclampsia, placental abruption, and stillbirth (Burton and Jauniaux, 2018; Pijnenborg et al., 2006; Brosens et al., 2011). Additionally, angiogenic imbalance, characterized by excess anti-angiogenic factors such as soluble fms-like tyrosine kinase-1 (sFlt-1) and soluble endoglin (sEng) alongside reduced levels of placental growth factor (PlGF) and vascular endothelial growth factor (VEGF), drives maternal endothelial dysfunction and contributes to hypertension, proteinuria, and further placental insufficiency (Levine et al., 2004; Zeisler et al., 2016; Maynard et al., 2003; Verlohren et al., 2012). Placental development is impacted by both maternal and fetal factors, including the maternal immune system. Excessive inflammation, complement activation, and altered interactions among uterine natural killer cells, macrophages, and T cells can impair trophoblast migration and vascularization, all of which predispose the mother to preeclampsia, FGR, and pregnancy loss (Redman and Sargent, 2010; Abrahams et al., 2005; Laresgoiti-Servitje, 2013). Moreover, placental cellular stress dysregulates apoptosis and other cell death pathways, damaging villous tissue and promoting syncytial shedding, creating a positive feedback loop of continued angiogenic imbalance and systemic inflammation in the mother (Burton et al., 2009; Heazell et al., 2011). Collectively, these mechanisms result in placental insufficiency, in which the placenta cannot adequately deliver oxygen and nutrients, leading to adverse outcomes such as intrauterine growth restriction, prematurity, or fetal demise (Kingdom and Kaufmann, 1997).

There are several long-term impacts of placental development issues. As mentioned, placental development issues result in a predisposition to preeclampsia with increased risk of cardiovascular risk (Bellamy et al., 2007; Kumar et al., 2022; Andrus and Wolfson, 2010), chronic hypertension (ACOG, 2019; Dubov et al., 2017), organ damage (Turbeville and Sasser, 2020), and neurological issues (Miller and Vollbracht, 2021). Whereby for the fetus, impaired placental function and fetal undernutrition, increase the offspring’s risk of cardiometabolic disorders in adulthood, including hypertension, coronary heart disease, and type 2 diabetes (Barker, 1995; Gluckman et al., 2010; Hanson and Gluckman, 2014).

## Impact of SSRI use during pregnancy

Understanding the implications of SSRI use during pregnancy needs careful consideration of both potential adverse fetal outcomes and the effects of untreated maternal depression. While SSRIs may pose certain risks to the developing fetus, withholding treatment for maternal depression can also result in significant adverse outcomes for both mother and child. Within the pregnant population, 8%–13% of pregnant women are prescribed SSRIs (Velasquez et al., 2013; Boone et al., 2025). Selective serotonin reuptake inhibitors work by targeting serotonin reuptake receptors SLC6A4, which genetically codes for SERT, allowing 5-HT to remain in the post-synaptic area or circulate in the blood (Baković et al., 2021; Sangkuhl et al., 2009). Increased extracellular 5-HT in the postsynaptic area is not uniformly beneficial in placental or fetal contexts, as serotonergic effects depend on developmental timing, anatomical compartments such as the maternal circulation, trophoblast, or fetal brain, and receptor subtype (Bonnin and Levitt, 2011). Serotonin is critical for placental development and fetal neurodevelopment; altered serotonin availability in the maternal compartment may produce divergent effects depending on the timing of these changes (Bonnin and Levitt, 2011; Oberlander et al., 2009). Therefore, increased serotonergic signaling cannot be assumed to be inherently protective or harmful, and its impact on placental and fetal outcomes is still incompletely defined in humans. Based on previous research and an understanding of SSRI impact on increased vasoconstriction resulting in downstream impacts on the offspring due to changes in hemodynamics and nutrient exchange (Brenner et al., 2007; Domingues et al., 2023a). Both pharmaceutical inhibition and genetic impairment of SERT may influence SERT activity. Research has demonstrated that mutations in the SERT-gene promotor may cause a potential vulnerability factor, specifically in the s-allele (Mandelli et al., 2007), resulting in an increased risk of major depressive episodes in the early postpartum period (Binder et al., 2010). More research is needed, though, to better understand the extent and mechanisms of these changes.

### Impact on mothers

In pregnant patients with moderate–severe depression, continuing an SSRI reduces the chance of symptomatic relapse and preserves day-to-day functioning. Previous research has shown that a prospective cohort study found relapse in approximately 68% who discontinued vs. approximately 26% who continued their medication (Boone et al., 2025). Concurrently, professional guidance emphasizes individualized treatment because untreated illness itself raises risks across pregnancy and the postpartum period (Boone et al., 2025; Cohen et al., 2006; ACOG, 2023; Yonkers et al., 2011). Additionally, maternal adverse events have been reported: late-pregnancy SSRI/SNRI exposure is associated with a small absolute increase in postpartum hemorrhage—likely *via* platelet 5-HT effects—most clearly shown in a large U.S. cohort, with multiple reviews concluding the excess risk is modest (Palmsten et al., 2013; Lupattelli et al., 2014; Sal et al., 2008). Evidence for hypertensive disorders is mixed, but recent meta-analysis suggests a modest association with gestational hypertension/preeclampsia that may be confounded by illness severity and other risk factors; clinicians should assess baseline preeclampsia risk when counseling (Palmsten et al., 2013; Lupattelli et al., 2014; Sal et al., 2008). During the postpartum period, effective SSRI treatment reduces recurrence of major depression and supports recovery and bonding; in high-risk women with prior postpartum depression, randomized data show sertraline prophylaxis substantially reduces relapse and delays time to recurrence *versus* placebo (Wisner et al., 2004). Overall, contemporary obstetric guidance supports access to SSRIs during pregnancy and lactation when indicated, balancing modest medication-related risks against the substantial maternal harms of undertreated depression (ACOG, 2023). Importantly, as described herein, studies examining SSRI use during pregnancy focus on classical pregnancy complications and depression; however, the physiological impact on both the mother and fetus during gestation has not been well examined or researched. This avenue of research could be beneficial in allowing the continued use of SSRI during pregnancy.

### Impact on fetus

Prenatal SSRI exposure has been studied extensively in the field of researching autism-like outcomes (Upadhyaya et al., 2023; Lebin and Novick, 2022; Castro et al., 2016; Ornoy and Koren, 2018; Uguz, 2018; Leshem et al., 2021; Hartwig et al., 2022), rather than the long-term impacts of *in utero* exposure to SSRIs. These studies have shown a pattern of small, mostly non-causal risks rather than large, consistent harms, with previous research finding no robust increase in overall major congenital malformations after accounting for underlying maternal illness. Although one SSRI (paroxetine) has been linked to a slightly higher risk of certain cardiac defects when used in the first trimester (Lebin and Novick, 2022; Huybrechts Krista et al., 2014). Late-pregnancy SSRI exposure has been associated with an increased risk of persistent pulmonary hypertension (PPHN) of the newborn in some studies, but the absolute risk remains small (Bérard et al., 2017). Infants exposed in utero—particularly with third-trimester exposure—may develop neonatal adaptation syndrome (Ornoy and Koren, 2018; Ornoy and Koren, 2017; Ornoy and Koren, 2019) (jitteriness, respiratory distress, poor tone, feeding/sleep problems); estimates vary, but many studies report transient symptoms in roughly 10%–30% of exposed newborns that typically resolve over days–weeks with supportive care (Brumbaugh et al., 2023). Observational links between prenatal SSRI use and outcomes such as preterm birth (Bandoli et al., 2020) or low birth weight (Li et al., 2020) have been hypothesized, but these associations are modest, and may be confounded by the effects of maternal depression itself (Cantarutti et al., 2016). Finally, the literature tying prenatal SSRI exposure to long-term neurodevelopmental disorders (autism spectrum disorder (Morales et al., 2018), ADHD (192), cognitive deficits (Leshem et al., 2021)) is mixed (Uguz, 2018; Suarez et al., 2022). Current hypotheses indicate possible associations, but higher-quality analyses, including sibling-comparison and meta-analyses (Hartwig et al., 2022), indicate that much of the signal is explained by confounding maternal illness, genetics, and environment rather than an apparent causal drug effect (Upadhyaya et al., 2023; Lebin and Novick, 2022).

## Gaps in literature

Despite extensive research, the impact of SSRI use during pregnancy remains unclear, with contradictory findings on safety, fetal development, and maternal outcomes, compounded by limited documentation of contributing variables. Previous studies have shown a significant increase in SSRI prescriptions with a pattern of doubling every 10 years since the 1980s. There was a significant increase in SSRIs usage from 2013 to 2023, with a spike occurring post March 2020 both in the US (Levin et al., 2022; Alsultan, 2024) and globally (Frangou et al., 2023; Otter et al., 2024). Importantly, data collected from the CDC showed that women were twice as likely to take SSRIs in comparison to men, which was observed for all of the age groups (Elgaddal et al., 2025) and irrespective of marital status (Brody and Gu, 2020).

Mechanistic evidence linking maternal SSRI exposure to placental dysfunction in humans is limited, with most of our understanding derived from animal studies (Bonnin and Levitt, 2011; Domingues et al., 2022a; Domingues et al., 2022b; Domingues et al., 2023b). Few investigations have explored the impact of long-term SSRI use prior to conception, despite the large population of younger females (Harris, 2024) being prescribed SSRIs increasing over time and the potential for cumulative effects on maternal vascular health and placental epigenetics (Lebin and Novick, 2022; Hutchison et al., 2018; Leshem et al., 2021; Ornoy and Koren, 2017). Evidence is also inconsistent due to the heterogeneity of the population prescribed these medications, as dosing is individualized based on each patient’s needs and demographic characteristics rather than being uniformly administered (Lebin and Novick, 2022; Domingues et al., 2022a). This variability complicates exposure definitions, outcome measures, and confounder control, placing research at a disadvantage. In particular, the interaction between advanced maternal age and SSRI exposure remains underexplored, despite both factors independently influencing placental efficiency through overlapping biological pathways (Lean et al., 2017; Qi et al., 2022).

Over the last couple of decades, there has been an increase in delayed childbearing, with a higher number of women entering pregnancy in their mid to late 30s or beyond (10% in 2016 to 12.5% in 2023) (Brown et al., 2025). Importantly, 1^st^ pregnancies at age 30 and above have increased from 22.3% (2016) to 25.1% (2023), indicating that 37.6% of 1^st^-time mothers are 30 years or older (Brown et al., 2025). Upon turning 35, any pregnancy falls into the category of an AMA pregnancy (Frick, 2021). Within this category, pregnancies are associated with possible increases in reproductive and obstetric risk primarily due to age-dependent changes in oocyte biology, with secondary contributions from uterine, placental, and systemic maternal factors. Previous research has shown that in women with AMA there are possible changes in the oocyte proteome, specifically lower levels of proteasome and key regulators of proteostasis and meiosis (Galatidou et al., 2024). Another contribution to the possible decline in fertility, aside from insufficient ovarian activity, is changes to the endometrium throughout the aging process. With age, the endometrium becomes thinner, and the uterus can shrink, depleting the endometrial blood supply (Marti-Garcia et al., 2024). Interestingly, there is a shortening of the menstrual cycle (Sebastian-Leon et al., 2025), changes to hormones such including reduced levels of early hCG (Chen et al., 2010), progesterone (Troisi et al., 2008), AMH (Dykgraaf et al., 2023), and estrogen (Troisi et al., 2008), as well as increased baseline FSH (Thum et al., 2008), alongside less responsive uterine muscles which impact labor (Arrowsmith et al., 2012). As for the placenta, in AMA pregnancies there are increased possibilities for premature senescence, reduced efficiency, and increases oxidative stress (Chen et al., 2021). Finally, women conceiving later on in life may have increased chances of pregnancy complications, including hypertension (Cooke and Davidge, 2019), gestational diabetes (Frick, 2021), postpartum hemorrhaging (Lao et al., 2014), caesarean sections (26.01% in AMA pregnancy vs. 15.26% in non AMA deliveries) (Veenstra et al., 2024; Braggion et al., 2023), and preterm birth (Cleary-Goldman et al., 2005). All these biological changes and pregnancy complications may contribute to the difficulties associated with AMA pregnancies.

This trend of waiting until later in life to get pregnant has been attributed to multiple social and socioeconomic factors, including romantic, financial, and career considerations (Datta et al., 2023). Many of these individuals also turn to assisted reproductive techniques due to decreases in fertility, complications with getting pregnant, or complications due to their own health (Jain and Singh, 2023). This complex and at times difficult journey can be emotionally and physically taxing, resulting in around 28%–44.32% of infertile women having depression (Kiani et al., 2021; Dawid et al., 2025). Up to 11% of this population is on an SSRI prescription to improve the health of the mother (Domar et al., 2013; Dawid et al., 2025). Overall, it should be noted that there is a global trend of women falling into the AMA category, with an increased occurrence of postpartum depression, with studies resulting in a 1.49 (Acheanpong et al., 2022) to 3.72 (Muraca and Joseph, 2014) fold increase in incidences of postpartum depression. In parallel to increased maternal age, studies have shown that rates of depression in AMA pregnancies increased from 8.9% (Ko et al., 2017) in 2012 to 10.8% (Bauman et al., 2020) in 2018. The increase in AMA, increased risk of postpartum depression, and the prescribing of SSRIs for the mothers’ mental health could create several critical factors that may simultaneously impact pregnancy yet remain untested or researched in humans. Advanced maternal age is associated with alterations in placental structure and function, including reduced vascularization and nutrient transport (Lean et al., 2017; Qi et al., 2022; Frick, 2021; Frederiksen et al., 2018; Patel et al., 2017), while SSRIs can affect 5-HT-mediated signaling pathways that regulate placental development. The combined effects of these factors may be additive or synergistic on fetal growth and pregnancy outcomes. Still, current research has not yet clarified these mechanisms, leaving a critical gap in understanding how maternal age and pharmacologic exposure intersect to influence placental and fetal health.

Although this review focused on the impact of SSRI use during pregnancy and its known effects on both maternal and fetal health, it is important to note that patients are often faced with a difficult choice: either discontinue their antidepressants to potentially reduce the risk of pregnancy complications (Liu et al., 2022) or continue medication with informed consent despite the associated risks (Lebin and Novick, 2022). This presents a challenging—and at times almost impossible—decision for expecting mothers. Therefore, more research is needed into therapeutic options such as blocking SERT receptors (5-HT2a/c receptors) (Bhat et al., 2023; Bolte et al., 2001). These receptors have already been investigated for the treatment of hypertension and preeclampsia in patients in order to mitigate the negative impact of SSRI use on placental vascularization, while allowing continued function of SSRIs in the neuroendocrine system. In previous research focused on patients with hypertension, it was shown that intravenous or oral ketanserin reduced blood pressure while mostly preserving the uteroplacental and fetal hemodynamics (Bolte et al., 2001; Hulme and Odendaal, 1986). Other research hypothesized it was due to decreasing serotonin-driven aggregation and endothelial dysfunction which may contribute to either of these conditions (Steyn and Odendaal, 2000). Therefore, with these previous findings alongside the findings of increased vasoconstriction and resistance in placentas of mice exposed to SSRIs (Domingues et al., 2023a), we hypothesize that this may a direction of possible therapeutic treatment.

In summary, placental health and pregnancy outcomes are influenced by both placental development and maternal biological factors. AMA (≥35 years) directly affects placental development and its adaptive capacity. Beyond that, exposure to selective serotonin reuptake inhibitors (SSRIs) can modify 5-HT signaling, which is critical for placental function, as the timing of placental development relies on this and other factors. Pregnancies in these contexts are associated with an increased risk of adverse outcomes and complications. This review highlights the importance of research that clarifies the underlying mechanisms associated with both maternal age and SSRI exposure, evaluates long-term outcomes, and explores targeted therapeutic strategies to support maternal mental health and fetal wellbeing. Additionally, it attempts to align the importance of interpretation of findings in mouse models to those in human studies.
